# Efficacy of allogenous fascia lata grafts in the management of lower eyelid retraction

**DOI:** 10.1007/s10792-023-02873-1

**Published:** 2023-09-18

**Authors:** Julia Prinz, Kathi Hartmann, Filippo Migliorini, Karim Hamesch, Peter Walter, Matthias Fuest, David Kuerten

**Affiliations:** 1 Department of Ophthalmology, RWTH Aachen University, Pauwelsstraße 30, 52074 Aachen, Germany; 2 Department of Orthopedics, RWTH Aachen University, Aachen, Germany; 3 Department of Gastroenterology and Hepatology, RWTH Aachen University, Aachen, Germany

**Keywords:** Lower eyelid retraction, Lower eyelid, Fascia lata, Graves’ disease, Trauma

## Abstract

**Purpose:**

To report on the use of allogenous fascia lata (FL) grafts in patients with lower eyelid retraction (LER).

**Methods:**

In this retrospective study, a consecutive series of 27 patients (39 eyes) with LER who underwent lower eyelid elevation with FL was included. Examinations including measurement of the palpebral fissure vertical height (PFVH), the inferior scleral show distance, the margin reflex distance 2 (MRD 2), and the evaluation of conjunctival hyperemia were conducted at baseline and after a mean postoperative time of 25.9 ± 25.5 (5.0–81.0, median 13.0, last follow-up) months in all patients.

**Results:**

At the last follow-up, a significant reduction of the PFVH (11.3 ± 1.7 *versus* 12.8 ± 2.1 at baseline, *p* < 0.001), the inferior scleral show distance (0.7 ± 1.0 mm *versus* 2.1 ± 1.1 at baseline, *p* < 0.001), and the MRD 2 (6.4 ± 0.9 *versus* 7.8 ± 1.3 at baseline, *p* < 0.001) occurred. The conjunctival hyperemia grading score (McMonnies) was significantly reduced (1.8 ± 0.7) at the last follow-up compared to baseline (2.6 ± 0.6, *p* < 0.001). No case of ectropion or entropion was observed at the last follow-up visit.

**Conclusion:**

In this case series, lower eyelid elevation with FL grafts as a spacer led to a significant reduction of the PFVH, MRD 2, inferior scleral show distance, and conjunctival hyperemia. No severe surgery-related complications occurred.

**Supplementary Information:**

The online version contains supplementary material available at 10.1007/s10792-023-02873-1.

## Introduction

Lower eyelid retraction (LER) caused by cicatricial changes is a common condition in patients with Graves’ orbitopathy (GO) as well as following trauma or prior lower eyelid blepharoplasty [1–3]. LER with lagophthalmos potentially leads to destructive ocular surface diseases, such as exposure keratopathy, corneal ulcer, and loss of vision [4]. Anatomically, LER results from a vertical shortening of the posterior lamella [5]. The correction of LER involves graft or non-graft surgical techniques [6–10]. Generally, non-graft techniques including recession, tenotomy, or extirpation of the capsulopalpebral fascia and inferior tarsal muscle, are used in mild cases of LER [2, 11, 12]. Numerous autologous grafts, including auricular cartilage [13], dermis fat [14], hard-palate mucosa [11], and spacer materials, such as porous polyethylene [15–17], have been used for more severe cases of LER [18]. Generally, the rationale behind grafts is to prevent re-scarring and to push the lower eyelid upwards [19]. However, harvest site morbidities pose a major limitation to autologous grafts [11]. Allogenous donor grafts, such as scleral grafts, are of limited availability and generally associated with a risk of transmitting the Human Immunodeficiency Virus (HIV) or slow viruses [2].

Fascia lata (FL) is the deep fascia of the thigh transmitting mechanical forces of the musculoskeletal system of the lower extremities [20]. FL mainly consists of a collagen matrix, fibrocytes, and fibroblasts [20, 21]. Its suitability for grafting has been attributed to the relative acellularity, low nutritional demands, tensile strength, and pliability [21, 22]. Allogenous FL with similar properties to autologous FL is commercially available and avoids patient’s donation site morbidity [23]. It is prepared using low dose gamma irradiation with sterilization of microbes [23]. Due to their advantages, FL grafts have been used in different surgical specialties, including urologic [24], orthopedic [25], neuro- [26], and general surgery [27]. In 1909, Payr pioneered the use of FL in ophthalmic surgery for the correction of congenital ptosis [28]. Since then, FL has been used for the treatment of numerous ophthalmic conditions, such as orbital implant extrusion, scleromalacia perforans, cicatricial entropion, and large vertical squint angles in patients with Graves’ orbitopathy [21, 29, 30].

In this study, we evaluated the use of allogenous FL as a graft for lower eyelid elevation in patients with LER.

## Material and methods

In this retrospective study, we included a consecutive series of 39 eyes of 27 patients with LER who underwent lower eyelid elevation with FL between December 2014 and May 2021. Thirty-four eyes of 23 patients with GO (87.2%), 3 eyes of 2 patients (7.7%) with iatrogenic LER following lower eyelid blepharoplasty, and 2 eyes of 2 patients (5.1%) following trauma (orbital floor fracture) were included. All patients were treated at the Department of Ophthalmology, RWTH Aachen University. Subjects included in this analysis were aged 18 years or older and were diagnosed LER due to GO, trauma, or postoperative/iatrogenic LER. Patients with involutional LER were excluded from our study. Patients with manifest vertical squint and patients following inferior rectus muscle elongation were also excluded.

**Fig. 1:**
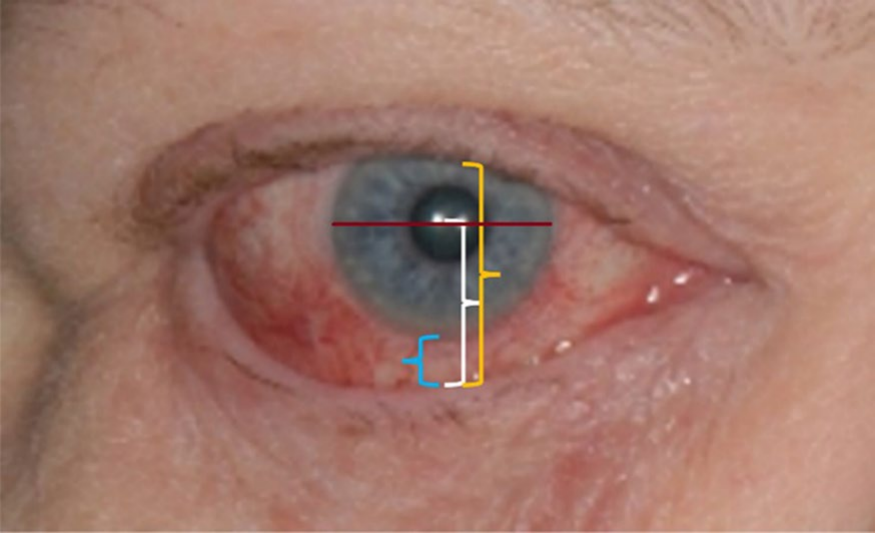
Measurement of the palpebral fissure vertical height (PFVH, yellow), the margin reflex distance 2 (MRD 2, white), and the inferior scleral show distance (blue). The parameters were calculated based on the assumption that the horizontal corneal diameter was 11.5 mm (red)

Ophthalmic examinations including photographs for measurement of the inferior scleral show distance, the margin reflex distance 2 (MRD 2) [31], the palpebral fissure vertical height (PFVH), and conjunctival hyperemia were conducted at baseline and after a mean postoperative time of 25.9 ± 25.5 (5.0–81.0, median 13.0, last follow-up) months in all patients. For the photographs, a light source was placed 50 cm before the patient’s face and one eye was occluded. The patient was then asked to fixate the light, providing the light reflex to be in the center of the pupil. Thus, the eye was photographed in the primary position. The MRD 2 was calculated as the distance from the corneal light reflex to the center of the lower eyelid margin (Fig. 1) [31]. The PFVH was measured as the distance between the upper and lower eyelid in vertical alignment through the center of the pupil (Fig. 1). The parameters were calculated based on the assumption that the horizontal corneal diameter was 11.5 mm as reported previously (Fig. 1) [32]. Conjunctival hyperemia was evaluated according to a grading scale with six grades (0–5) introduced by McMonnies for contact lens wearers [33]. The scale considered the number, density, and tortuosity of conjunctival vessels [33].

All values were measured by two independent investigators blinded to the surgical status. Disagreements between the authors were debated and resolved by a third author.

The study adhered to the tenets of Helsinki. It was approved by the medical ethics committee of the RWTH Aachen (EK 465/21).

### Surgical technique

**Fig. 2:**
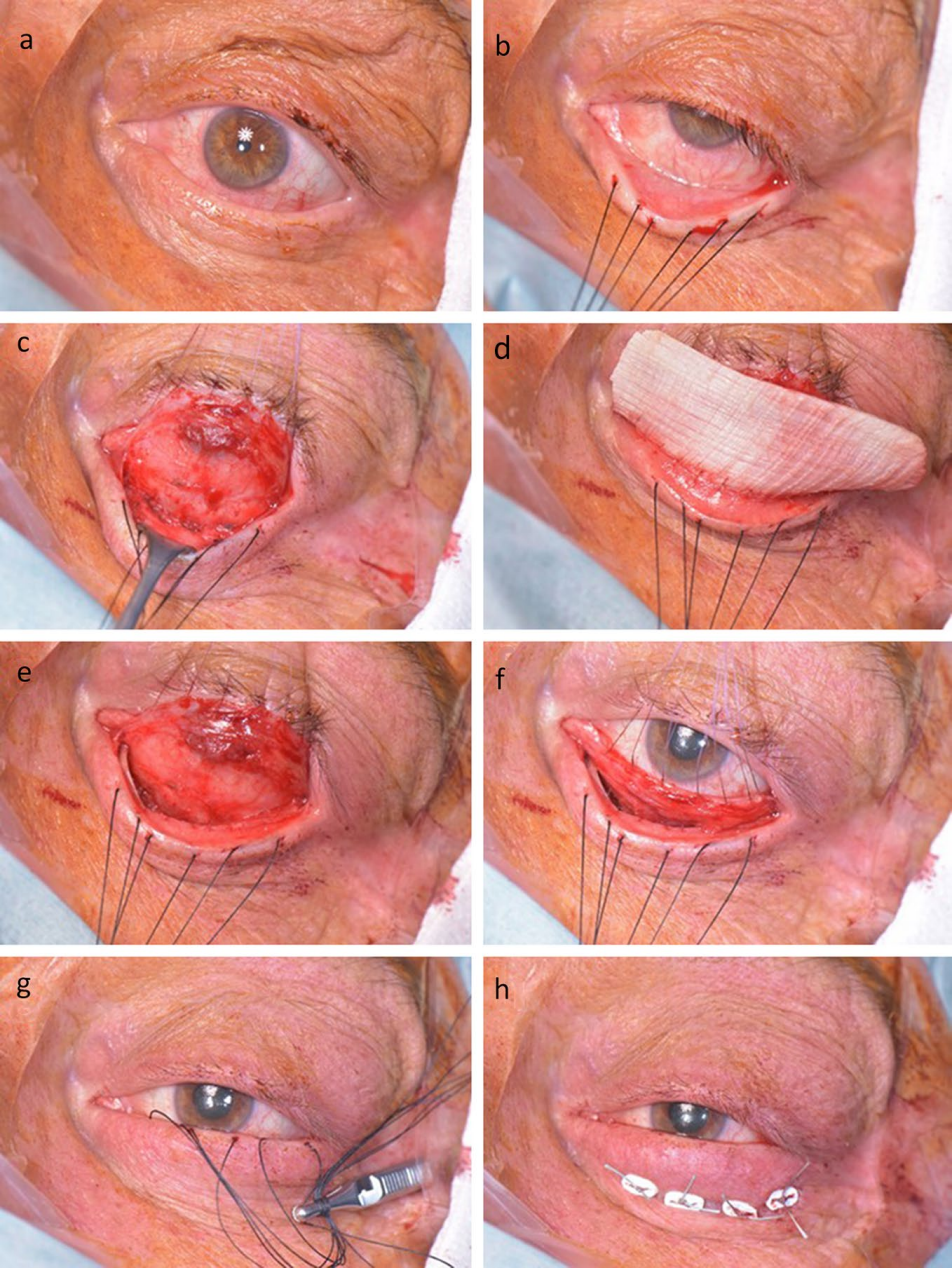
Intraoperative photographs with the preoperative situs (**a**) and the lower eyelid secured by retractor sutures (4–0 silk sutures, Ethicon, Johnson and Johnson Medical GmbH, Norderstedt, Germany). Following a xylocaine infiltrative anesthesia to separate the lower eyelid tissue layers, the lower fornix conjunctiva was incised along its entire length at the level of the inferior edge of the tarsus (**c**). Afterward, the inferior edge of the conjunctiva was secured with Vicryl 8–0 (Ethicon) sutures. The conjunctiva was dissected downwards toward the orbital floor (**c**). The FL graft was cut to the whole width of the eyelid with an overlap of 2–3 mm laterally and nasally. It was sutured with Vicryl 8–0 to the exposed lower edge of the tarsus (**d**). Then, the FL graft was inserted into the previously prepared gap between the conjunctiva and the lower eyelid retractors (**e**,**f**). The conjunctiva was re-adapted (**g**). Finally, 4 anterior–posterior penetrating traction sutures (Sulene USP 2, Serag-Wiessner, Naila, Germany) were performed below the tarsus and tied to the skin with foam bolsters (h)

The aim of each surgery was the reduction of LER by elevation of the lower eyelid. All procedures in this study were performed by the same experienced surgeon (K.H.). Surgery was performed under general anesthesia. Allogenous Tutoplast FL (Bess Medizintechnik GmbH, Berlin, Germany) was used as graft material. The FL graft was first rehydrated in balanced salt solution (Alcon BSS, Alcon Pharma GmbH, Freiburg, Germany). The lower eyelid was secured by retractor 4–0 silk sutures (Ethicon, Johnson and Johnson Medical GmbH, Norderstedt, Germany, Fig. 2b). After xylocaine infiltrative anesthesia to separate the lower eyelid tissue layers, the lower fornix conjunctiva was incised along its entire length at the level of the inferior edge of the tarsus (Fig. 2c). Then, the inferior edge of the conjunctiva was secured with Vicryl 8–0 (Ethicon, Johnson and Johnson Medical GmbH, Norderstedt, Germany) sutures. With the help of the traction sutures and the Vicryl sutures, the inferior edge of the conjunctiva and the lower eyelid edge can be held apart under tension. As the lower edge of the orbit should be palpable through the conjunctival incision, the conjunctiva was dissected downwards toward the orbital floor (Fig. 2c). The lower edge of the orbit was palpated for any additional scars or tension that may need to be released.

The FL graft was cut to the whole width of the eyelid with an overlap of 2–3 mm laterally and nasally. Afterward, it was sutured with Vicryl 8–0 to the exposed lower edge of the tarsus (Fig. 2d). The FL graft was then inserted into the previously prepared gap between the conjunctiva and the lower eyelid retractors (Fig. 2e). The fibers of the FL graft were transversely orientated (Fig. 2). Then, the upper and the lower incision edges of the conjunctiva were re-adapted with Vicryl sutures, which were initially put in place (Fig. 2g). This way, the FL graft was completely covered. Finally, 3 to 4 anterior–posterior penetrating traction sutures (Sulene USP 2, Serag-Wiessner, Naila, Germany) were performed below the tarsus (Fig. 2h). The Sulene sutures were tied to the skin with foam bolsters protecting the skin and avoiding a dislocation of the graft. These sutures were used to prevent scarring with a downward traction effect, and they were removed 2 weeks postoperatively. Intraoperatively, a moderate over-effect was desired.

### Statistical analysis

Statistical analysis was performed using the Statistical Package for Social Sciences (IBM SPSS Statistics for Windows, Version 25, Armonk, NY: IBM Corp.). All values are displayed as mean ± standard deviations (SD). Means for continuous variables were compared using paired T tests. Chi-square test and Fisher’s exact test were used for categorical variables. A p value of < 0.05 was considered statistically significant.

## Results

### Patients’ characteristics

Our study included 21 right (53.8%) and 18 left eyes of 27 patients (12 bilateral surgeries). The mean age of all patients was 56.3 ± 9.9 (34.0–71.0) years. Ten patients (37.0%) were male.

Prior to lower eyelid elevation, 8 patients (14 eyes) with GO underwent orbital decompression. Previously, bilateral recession of the lateral rectus muscles was performed in 1 patient with GO. Another patient with GO previously underwent bilateral levator lengthening by marginal myotomy. A patient with LER following trauma received a prior inferior rectus muscle adhesiolysis. The mean Clinical Activity Score [34] in the GO patients was 1.5 ± 0.7 at the time of lower eyelid elevation and was not significantly changed at the last follow-up (1.6 ± 0.6, *p* = 0.293).

**Fig. 3:**
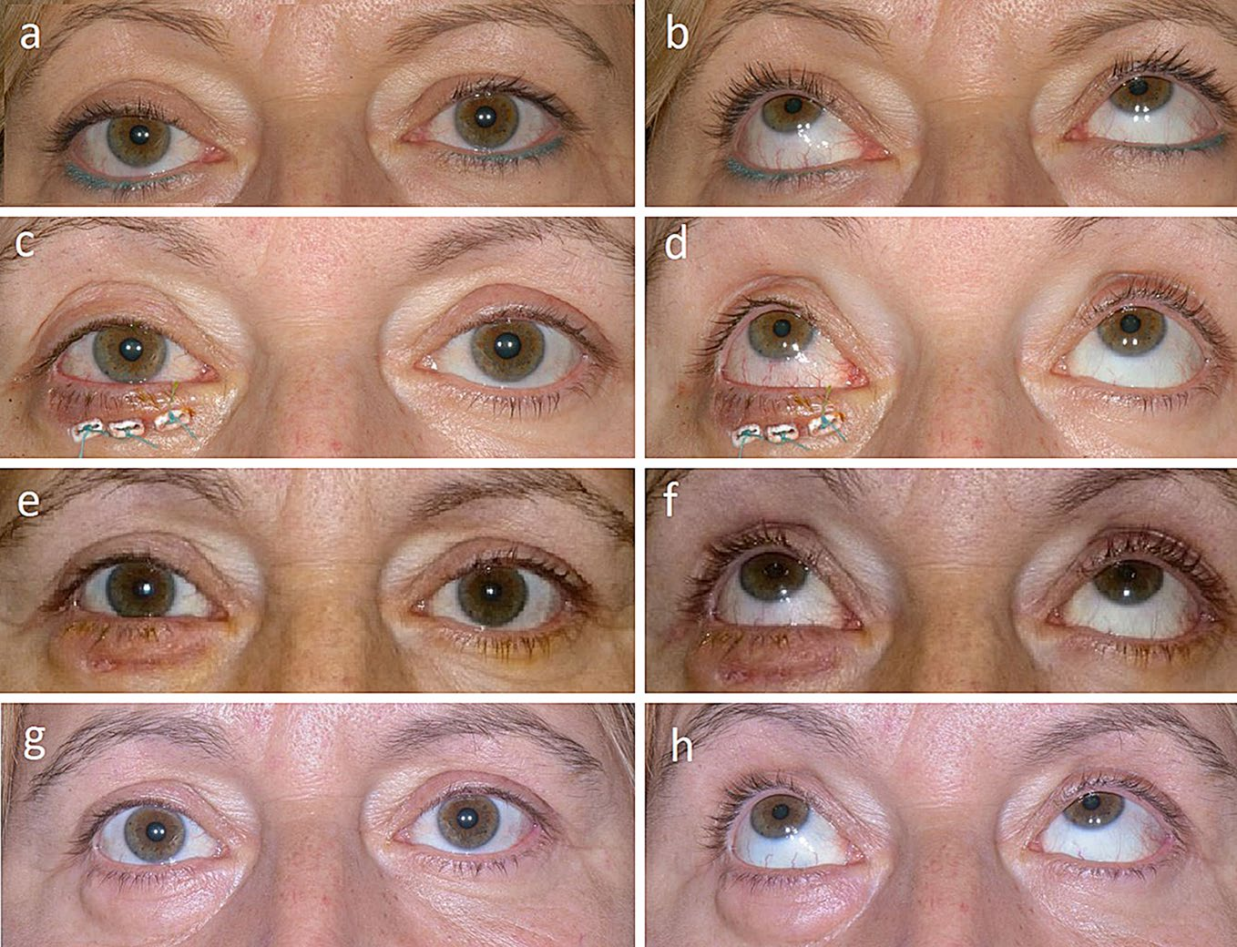
Appearance of a 53-year-old female patient (patient number 5 according to supplementary Table 1) undergoing lower eyelid elevation with fascia lata. The patient previously underwent bilateral orbital fat decompression and lateral tarsal strip surgery 12 months ago. At baseline, a lower eyelid retraction (LER) was witnessed at the right eye in primary position (PP, **a**) and upgaze (**b**). At the first postoperative day, a slight over-effect due to lower eyelid swelling was witnessed in PP (**c**) and upgaze (**d**) with the foam bolsters in situ. At two weeks postoperatively, the sutures were removed and a slight residual swelling of the lower eyelid was observed in PP (**e**) and upgaze (**f**). The postoperative appearance at the 3-month follow-up is displayed in PP (**g**) and upgaze (**h**) with residual lower eyelid swelling, which regressed completely until the 6-month follow-up (no photographs)

A significant reduction of the inferior scleral show distance (0.7 ± 1.0 mm *versus* baseline 2.1 ± 1.1, *p* < 0.001), MRD 2 (6.4 ± 0.9 *versus* baseline 7.8 ± 1.3, *p* < 0.001), and PFVH (11.3 ± 1.7 *versus* baseline 12.8 ± 2.1, *p* < 0.001) occurred at the last follow-up (Table 1, Supplementary Table 1). The conjunctival hyperemia grading score was significantly reduced at the last follow-up (1.8 ± 0.7) compared to baseline (2.6 ± 0.6, *p* < 0.001). No case of ectropion or entropion was witnessed at the last follow-up. Figure 3 shows the pre- und postoperative findings of a patient undergoing lower eyelid elevation with FL.

**Table 1:** Inferior scleral show distance in mm, margin reflex distance 2 in mm, palpebral fissure vertical height in mm, and conjunctival hyperemia grading score according to McMonnies [28] at baseline and at the last follow-up (last FU) of 25.9 ± 25.5 (median 13.0) months. Compared to the baseline values, a significant reduction of all parameters occurred at last follow-up

Endpoint	Baseline	Last FU	*P *value
Inferior scleral show distance	2.1 ± 1.1	0.7 ± 1.0	< 0.001
Margin reflex distance 2	7.8 ± 1.3	6.4 ± 0.9	< 0.001
Palpebral fissure vertical height	12.8 ± 2.1	11.3 ± 1.7	< 0.001
Conjunctival hyperemia	2.6 ± 0.6	1.8 ± 0.7	< 0.001

### Complications

No intraoperative complications occurred. At the first postoperative day, a case of moderate hemorrhage at the surgery site most likely due to continued factor Xa inhibitor anticoagulation was reported in a 71-year-old female patient. The hemorrhage stopped spontaneously after 30 min of moderate wound compression. No further postoperative complications occurred.

## Discussion

In this study, lower eyelid elevation with FL was evaluated in 39 eyes of 27 patients with LER. Postoperatively, we found a significant reduction of the PFVH, inferior scleral show distance, and MRD 2. In addition, the conjunctival hyperemia grading score was significantly reduced at the last follow-up compared to baseline. The FL graft approximated the lower eyelid in contour and stiffness. No severe intra- or postoperative complications occurred with a mean follow-up of 25.9 ± 25.5 months. To the best of our knowledge, this is the first study addressing the outcomes of lower eyelid elevation with allogenous FL grafts.

Autogenous FL was initially used to correct LER by Flanagan and Campbell in 1981 [21]. Patients with GO, cicatricial entropium, and extruding orbital implants were included. The authors reported FL grafts to be effective and readily available. With a follow-up of 2 months to 3 years, no relevant surgery-related complications occurred [21]. However, autogenous FL graft harvesting extends the surgical procedure and leaves a scar on the thigh [11, 35]. A dose of 2 mm autogenous FL was reported by Flanagan and Campbell to yield 1 mm correction of LER (dose effect: 0.5 mm LER reduction per mm autogenous FL graft) [21]. In our study, no dose effects were calculated as the FL grafts were not fixed by sutures to the lower edge of the exposed conjunctiva nor to the capsulopalpebral fascia. Instead, the FL grafts were placed without sutures on the orbial floor to create a barrier for recurrent scarring.

In a study by Sendul et al., a lower eyelid sling technique with autologous FL resulted in a significant reduction of punctate epitheliopathy and successful lower eyelid repositioning in 10 patients with lagophthalmos due to facial paralysis [4]. No cases of exposure of the suspension material were reported. However, a patient developed postoperative wound infection at the harvest site [4]. Further complications associated with harvesting of autologous FL might include postoperative numbness, pain, hematoma, muscle herniation, superficial phlebitis, and scar formation at the harvest site [36, 37]. In contrast, allogenous FL grafts avoid harvest site morbidities [37]. To date, no relevant host reaction or absorption of the allogenous FL grafts have been reported, which minimizes the possibility of significant over- or under-correction of the LER [21, 30].

In our study, the MRD 2, PFVH, and inferior scleral show distance were measured in portrait photographs of the included patients. Generally, the MRD 2 is used to determine the amount of LER [31]. The normal average value of MRD 2 is 5.5 mm [31]. At baseline, the mean MRD 2 was 7.8 ± 1.3 mm in our cohort, and it was reduced to 6.4 ± 0.9 mm at the last follow-up. Thus, the significant reduction of MRD 2 shows the efficacy of lower eyelid elevation with FL in our study. The PFVH normally ranges between 7 and 12 mm [38]. At baseline, the mean PFVH (12.8 ± 2.1 mm) was elevated in our cohort. A significant reduction to a PFVH of 11.3 ± 1.7 mm occurred at the last follow-up. In addition, the conjunctival hyperemia score was significantly reduced at last follow-up. Conjunctival hyperemia is common in patients with dry eye disease due to lagophthalmos or GO [39].

The ideal graft for treating LER would mimic the tarsal-conjunctival tissue in pliability, resilience and thickness [40]. Previously, various techniques have been proposed for the correction of LER [11]. Non-graft techniques, such as free tenotomy or inferior retractor recession, only yielded limited success and therefore are only recommended for mild cases of LER [11]. Numerous graft materials have been used to treat LER, including auricular cartilage [13], sclera [2], dermis fat [14], and hard-palate mucosa [11]. The rationale for using grafts in the correction of LER is to lift the lower eyelid upwards and to prevent recurrent scarring due to their barrier function [19]. However, auricular cartilage is known to be stiff and rigid [41]. Therefore, its conformability on the globe is restricted [41]. Clinically, postoperative downgaze with auricular cartilage grafts might be impeded due to stiff, immobile lower eyelids [11]. Donor sclera is associated with recurrent retraction due to graft fibrosis [2]. Moreover, scleral grafts have a risk of transmitting HIV or slow viruses [2]. Acceptable results of LER correction using hard palate mucosa have been reported previously [11]. However, the principal concern of hard palate mucosa is donor site morbidity, mainly including hemorrhage, candidiasis and oro-nasal fistulae [11]. The major disadvantages of dermis fat grafts are surface keratinization and growth of hairs resulting in ocular surface complications [42].

With a mean follow-up of 25.9 ± 25.5 months, no postoperative complications, such as extrusion of the graft, scar formation, entropion or ectropion were witnessed in our study, which is attributable to the high biocompatibility of FL. To date, numerous benefits of allogenous FL grafts, such as a very low risk of infection, displacement, extrusion, harvest site morbidities or transmission of prion and viral diseases have been reported [43]. Previous histologic reports emphasize the high possibility of complete integration of FL tissue transplants into the body’s own tissue [43]. Generally, the tensile strength of the FL is particularly high in the direction of the fibers and only minimally transversely [44]. In lower eyelid elevation, the fibers of the FL graft were transversely orientated as the surgery aims to recess the lower lid retractors instead of transmitting forces.

The lack of a control group, the small sample size and the retrospective design of our study pose limitations for the analysis of lower eyelid elevation with FL. Future prospective studies are warranted to directly compare the outcomes of lower eyelid elevation with FL to those with other graft materials.

Altogether, in this case series, lower eyelid elevation with FL grafts as a spacer was associated with a significant reduction of the PFVH, MRD 2, inferior scleral show distance, and conjunctival hyperemia. No relevant surgery-related complications occurred. Intraoperatively, the FL grafts showed a high pliability and conformability to the globe. However, future studies are needed to directly compare the outcomes of lower eyelid elevation with FL to those with other autologous or allogenous grafts and to non-graft techniques.

### Supplementary Information

Below is the link to the electronic supplementary material.

Supplementary file1 (DOCX 27 KB)
