# Supplementary material for: Efficacy of allogenous fascia lata grafts in the management of lower eyelid retraction
Source: Int Ophthalmol. 2023 Sep 18;43(12):4729–37. doi: 10.1007/s10792-023-02873-1 (PMC10724318; doi:10.1007/s10792-023-02873-1)
Supplement: Supplementary file 1 — Supplementary file1 (DOCX 27 KB) [file 10792_2023_2873_MOESM1_ESM.docx]

|  | **Baseline** | | | | **Last FU** | | | | |
| --- | --- | --- | --- | --- | --- | --- | --- | --- | --- |
| number | PFVH  [mm] | MRD 2 [mm] | inferior scleral show [mm] | conjunctival hyperaemia | PFVH [mm] | | MRD 2 [mm] | inferior scleral show  [mm] | conjunctival hyperaemia |
| 1 | 14.2 | 8.5 | 3.2 | 4.0 | 11.3 | 5.9 | | 0.6 | 3.0 |
| 2 | 13.2 | 7.8 | 2.0 | 3.0 | 12.5 | 5.8 | | 0.0 | 2.0 |
| 3 | 14.3 | 7.5 | 1.0 | 3.0 | 14.0 | 6.9 | | 0.5 | 2.0 |
| 4 | 10.9 | 7.6 | 2.1 | 2.0 | 9.0 | 6.3 | | 0.7 | 1.0 |
| 5 | 11.5 | 8.1 | 2.0 | 2.0 | 11.0 | 6.4 | | 0.5 | 1.0 |
| 6 | 14.3 | 7.9 | 2.0 | 2.0 | 13.2 | 7.6 | | 1.7 | 2.0 |
| 7 | 15.0 | 8.7 | 2.9 | 2.0 | 12.7 | 6.5 | | 0.8 | 1.0 |
| 8 | 16.0 | 9.2 | 3.5 | 3.0 | 12.0 | 6.2 | | 0.5 | 1.0 |
| 9 | 14.8 | 8.8 | 3.4 | 2.0 | 13.5 | 7.5 | | 2.2 | 1.0 |
| 10 | 14.0 | 8.1 | 2.8 | 3.0 | 13.0 | 7.5 | | 2.3 | 1.0 |
| 11 | 13.0 | 8.0 | 2.8 | 3.0 | 12.0 | 7.5 | | 2.3 | 1.0 |
| 12 | 14.5 | 8.4 | 3.0 | 3.0 | 12.9 | 7.2 | | 1.8 | 2.0 |
| 13 | 13.4 | 6.9 | 1.8 | 3.0 | 11.2 | 4.6 | | -0.5 | 2.0 |
| 14 | 9.2 | 6.6 | 1.1 | 2.0 | 8.8 | 5.6 | | 0.0 | 2.0 |
| 15 | 15.4 | 7.6 | 1.8 | 2.0 | 12.2 | 4.6 | | -1.0 | 1.0 |
| 16 | 14.0 | 6.6 | 1.0 | 2.0 | 13.2 | 5.9 | | 0.3 | 1.0 |
| 17 | 12.0 | 8.6 | 2.6 | 2.0 | 10.0 | 6.0 | | 0.0 | 2.0 |
| 18 | 8.1 | 6.2 | 0.0 | 3.0 | 7.0 | 5.0 | | -1.0 | 2.0 |
| 19 | 10.8 | 6.0 | 0.5 | 3.0 | 10.1 | 5.5 | | 0.0 | 2.0 |
| 20 | 15.4 | 12.2 | 5.0 | 2.0 | 11.4 | 7.8 | | 0.5 | 2.0 |
| 21 | 15.4 | 10.0 | 3.9 | 2.0 | 13.6 | 7.9 | | 1.8 | 1.0 |
| 22 | 13.1 | 8.8 | 2.8 | 2.0 | 12.1 | 8.1 | | 2.1 | 2.0 |
| 23 | 12.1 | 7.9 | 2.5 | 2.0 | 11.1 | 7.4 | | 2.0 | 2.0 |
| 24 | 14.3 | 7.4 | 2.0 | 2.0 | 13.0 | 7.1 | | 1.6 | 2.0 |
| 25 | 14.8 | 6.6 | 1.1 | 2.0 | 12.2 | 6.5 | | 1.0 | 1.0 |
| 26 | 10.4 | 4.9 | -1.0 | 3.0 | 10.3 | 6.6 | | 0.7 | 2.0 |
| 27 | 11.7 | 6.7 | 1.5 | 3.0 | 10.3 | 5.9 | | 0.7 | 2.0 |
| 28 | 11.7 | 6.6 | 1.5 | 2.0 | 11.0 | 5.9 | | 0.8 | 2.0 |
| 29 | 10.5 | 7.2 | 1.8 | 2.0 | 10.5 | 7.5 | | 2.0 | 2.0 |
| 30 | 13.0 | 6.8 | 1.2 | 3.0 | 11.5 | 5.6 | | 0.0 | 1.0 |
| 31 | 14.5 | 8.0 | 2.8 | 3.0 | 13.3 | 7.4 | | 2.2 | 2.0 |
| 32 | 14.0 | 7.1 | 2.0 | 3.0 | 12.0 | 6.0 | | 1.0 | 2.0 |
| 33 | 12.7 | 8.9 | 3.2 | 4.0 | 8.9 | 5.7 | | 0.0 | 3.0 |
| 34 | 12.7 | 8.5 | 1.8 | 3.0 | 10.2 | 6.4 | | -0.5 | 2.0 |
| 35 | 11.0 | 6.7 | 1.2 | 3.0 | 11.5 | 6.9 | | 1.5 | 3.0 |
| 36 | 7.4 | 6.6 | 1.4 | 3.0 | 6.9 | 5.2 | | 0.0 | 3.0 |
| 37 | 12.0 | 8.5 | 3.4 | 3.0 | 11.5 | 5.6 | | 0.5 | 2.0 |
| 38 | 12.8 | 7.5 | 2.0 | 3.0 | 10.9 | 5.5 | | 0.0 | 3.0 |
| 39 | 10.2 | 8.3 | 2.1 | 4.0 | 9.4 | 5.8 | | -0.5 | 3.0 |

**Supplementary Table 1:** Values of the of palpebral fissure vertical height (PFVH) in mm, margin reflex distance 2 (MRD 2) in mm, inferior scleral show distance in mm, and conjunctival hyperaemia according to McMonnies [1] in 39 eyes at baseline and at the last follow-up (Last FU) of 25.9 ± 25.5 (median 13.0) months.

1. McMonnies CW, Chapman-Davies A (1987) Assessment of conjunctival hyperemia in contact lens wearers. Part I. Am J Optom Physiol Opt 64(4):246-250. doi:10.1097/00006324-198704000-00003
